# Transcriptomic evidence for versatile metabolic activities of mercury cycling microorganisms in brackish microbial mats

**DOI:** 10.1038/s41522-021-00255-y

**Published:** 2021-11-19

**Authors:** Adrien Vigneron, Perrine Cruaud, Johanne Aubé, Rémy Guyoneaud, Marisol Goñi-Urriza

**Affiliations:** 1 Université de Pau et des Pays de l’Adour, E2S UPPA, CNRS, IPREM, Pau, France; 2 Independent Researcher, Lourenties, France; 3 Present Address: Univ Brest, ifremer, CNRS, Laboratoire de Microbiologie des Environnements Extrêmes, 29280 Plouzané, France

**Keywords:** Water microbiology, Biofilms, Metagenomics

## Abstract

Methylmercury, biomagnifying through food chains, is highly toxic for aquatic life. Its production and degradation are largely driven by microbial transformations; however, diversity and metabolic activity of mercury transformers, resulting in methylmercury concentrations in environments, remain poorly understood. Microbial mats are thick biofilms where oxic and anoxic metabolisms cooccur, providing opportunities to investigate the complexity of the microbial mercury transformations over contrasted redox conditions. Here, we conducted a genome-resolved metagenomic and metatranscriptomic analysis to identify putative activity of mercury reducers, methylators and demethylators in microbial mats strongly contaminated by mercury. Our transcriptomic results revealed the major role of rare microorganisms in mercury cycling. Mercury methylators, mainly related to *Desulfobacterota*, expressed a large panel of metabolic activities in sulfur, iron, nitrogen, and halogen compound transformations, extending known activities of mercury methylators under suboxic to anoxic conditions. Methylmercury detoxification processes were dissociated in the microbial mats with methylmercury cleavage being carried out by sulfide-oxidizing *Thiotrichaceae* and *Rhodobacteraceae* populations, whereas mercury reducers included members of the *Verrucomicrobia*, *Bacteroidetes*, *Gammaproteobacteria*, and different populations of *Rhodobacteraceae*. However most of the mercury reduction was potentially carried out anaerobically by sulfur- and iron-reducing *Desulfuromonadaceae*, revising our understanding of mercury transformers ecophysiology.

## Introduction

Mercury (Hg) is a toxic and widespread heavy metal of natural and anthropogenic origins that accumulates in soils and sediments^1^. The methylmercury (MeHg), largely produced from methylation of inorganic mercury by anaerobic microorganisms^2^, is of special concern since this compound is highly toxic and biomagnifies in aquatic food webs, generating a health hazard to wild animals and humans^3^. Proteins associated with this metabolic capacity are coded by the gene pair *hgcAB*, initially detected in *Geobacter* and *Desulfovibrio* strains^4^ and now identified in genomes of few iron reducers, sulfate reducers, and methanogens^5^. Metagenomic mining and recovery of metagenome-assembled genomes (MAGs) of uncultured microbial populations also extended the phylogenetic and metabolic diversity of *hgcAB*-bearing lineages in various environments^6–8^, suggesting that the list of known methylmercury producers is far from complete. However, the mercury methylation activity of these uncultured lineages remains to be confirmed. Parameters driving *hgcAB* gene expression remain unknown. The characterization of cultured mercury methylators revealed that the expression of those genes is not inducible by mercury^9^, and no link have been observed between their expression and the methylation potential^10^. The expression of *hgcA* has been proposed to be constitutive but recent metatranscriptomic analysis of environmental samples challenged this hypothesis^6^. Therefore, further work is required to understand the in situ expression of *hgcAB* and, ultimately, the toxic methylmercury production.

Microbial activities that directly or indirectly affect Hg(II) methylation or MeHg degradation play a critical role in modulating mercury toxicity^11^. The prokaryotic mercury resistance mechanism, encoded by the *mer* operon plays a key role in this paradigm. Within the *mer* operon, *merA* gene encodes the mercuric reductase, reducing Hg(II) to gaseous mercury Hg(0)^11,12^, while *merB* gene encodes an alkylmercury lyase, cleaving organomercury compounds, including methylmercury^13,14^. The combinatorial action of *merA* and *merB* allows the complete detoxification of a broad spectrum of mercury compounds, providing a major decontamination mechanism for various microbial lineages in mercury contaminated environments. The taxonomic and functional diversity of these microorganisms remains unclear, blurred by numerous horizontal gene transfer events that frequently fractionated the *mer* operon in microbial genomes, but could be limited to aerobic microorganisms^11^. Furthermore, although the genetic capabilities of mercury cycling microorganisms have been previously investigated^6–8^, their gene expression profiles in situ remain largely unexplored, leaving their activities in natural habitat poorly understood.

The Etang de Berre in the south of France is the second largest European brackish lagoon covering 155.3 km^2^. The edge of the lagoon has a strong history of petroleum industry activity, leading locally to hydrocarbon pollutions and regionally to mercury atmospheric deposition. Shallow sediments of the lagoon are colonized by photosynthetic microbial mats. These microbial ecosystems are thick biofilms with a complex structural organization with filamentous cyanobacteria, autotrophic and heterotrophic prokaryotic lineages, protists, fungi, metazoans, and viruses distributed across the thickness and the step gradients of light, oxygen, nitrogen and sulfur compounds, which are characteristic of these biofilms^15^. Microbial mats play critical roles in many environments as hot spots of biological production, carbon mineralization, organic matter degradation, sediment stabilization, and decontamination of various pollutants including hydrocarbons or heavy metals^15–17^. The multiple environmental niches of these stratified biofilms gather a large variety of aerobic and anaerobic microbial activities, providing opportunities to better understand the complexity of microbial reactions and interactions within geochemical cycles. If environmental drivers of the microbial community structure of these mats have been previously analyzed^15^, microbial mercury transformations throughout the environmental gradients of the biofilm remain unexplored.

**Fig. 1:**
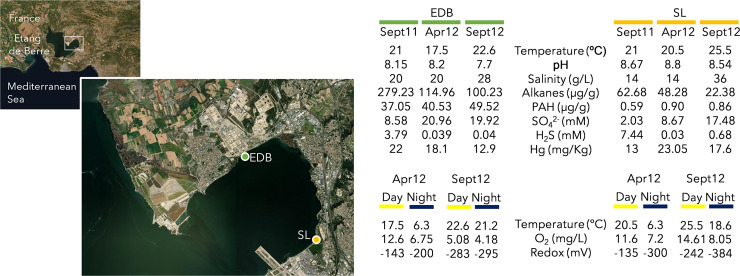
Description of the study sites. EDB and SL microbial mats, separated by 5 km, are both localized in the Etang de Berre area, South of France. EDB mat was collected near an oil industry wastewater discharge whereas SL mat was sampled in the Salins du Lion bird reserve. Chemical and physical parameters of the microbial mats during the sampling events. Alkanes include C11 to C38. PAH polycyclic aromatic hydrocarbons. Additional and more detailed dataset is available in Aubé et al. (2016, 2020)^15^^,^^19^.

In this study, we applied a genome-resolved metagenomics and metatranscriptomics approach to determine the taxonomy, functional potential, and putative activity of microbial mercury transformers in two microbial mats recovered from the Etang de Berre area, which are strongly contaminated by mercury (17.8 ± 4.1 μg of Hg per gram, Fig. 1) due to atmospheric deposition from neighboring industries and bioaccumulation of mercury by the microbial mats^18^. A total of 30 metagenomic datasets, covering the spatial and temporal variability of the microbial mats, was generated, leading to 389 GB of sequences, then co-assembled, resulting in the recovery of 407 high- to medium-quality MAGs. Mercury cycling populations were identified and protein sequences of these microbial MAGs were compared to 300 GB of rRNA-depleted metatranscriptomic sequences produced from the same samples. The expression profiles of *hgcA, merA, merB* genes, and energetical metabolic pathways were investigated at the population level, revealing a larger variety of metabolic activities than usually presumed for mercury cycling microorganisms. This study extends our knowledge on the physiology, the environmental niches, and the involvement in biogeochemical cycling of mercury-transforming microorganisms in coastal sediments.

## Results

### Geochemistry of the microbial mats

The taxonomic and genetic composition as well as the expression profile of two coastal microbial mats developing in neighboring brackish lagoons, located at Berre l’Etang (EDB) and Salins du Lion (SL) (Fig. 1) were investigated in fine detail. Detailed chemical and physical parameters of both microbial mats during the sampling events have been previously published^15,19^. As expected, both mats have high sulfate and sulfide concentrations while oxygen concentration and redox potential measurements indicated that both aerobic and anoxic niches likely cooccur in the microbial mats. Presence of alkanes and polycyclic aromatic hydrocarbons, coming from the oil industries, is the main environmental feature that distinguishes the two microbial mats (Fig. 1).

### Microbial community composition

**Fig. 2:**
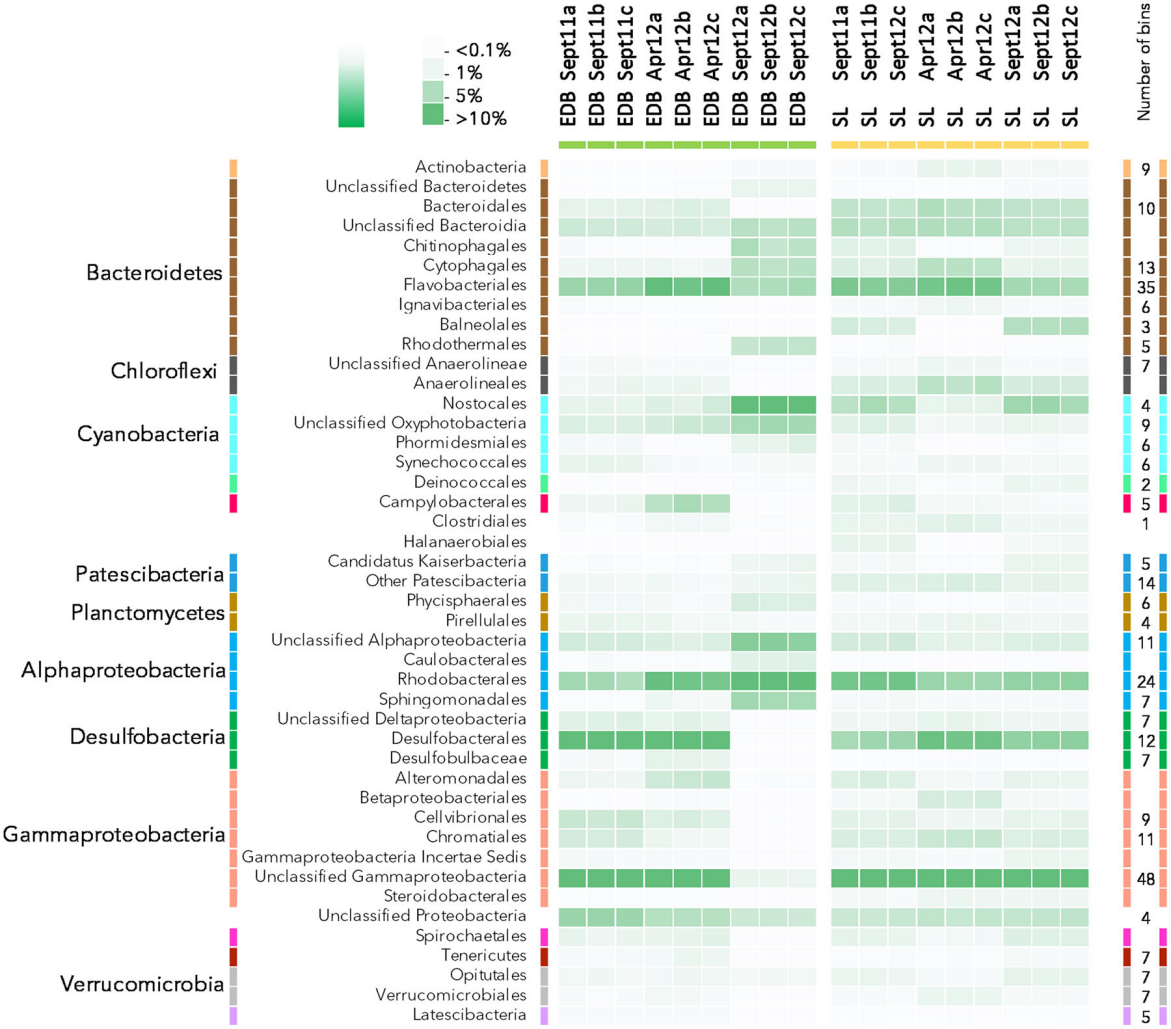
Microbial community composition of the microbial mats from Etang de Berre (EDB) and Salins du Lion (SL). Intensity of the color in the heatmap is proportional to the relative proportion of each group in the pool of 16S rRNA reads recovered from the metagenomic dataset. Only lineages representing more than 1% of the reads in at least one sample were represented. *Bacteroidetes* lineages are represented in brown, *Chloroflexi* in dark gray, *Cyanobacteria* in cyan, *Planctomycetes* in ochre, *Alphaproteobacteria* in blue, *Desulfobacterota* in green, *Gammaproteobacteria* in orange and *Verrucomicrobia* in gray. The number of bins associated with each taxonomic group is indicated at the right. 94 additional bins were recovered from lineages representing less than 1% of the 16S rRNA reads.

The microbial community composition was determined using all 16S rRNA genes recovered from the metagenomic datasets (2.1 ± 0.9 × 10^4^ 16S rRNA genes per sample; Fig. 2). A discontinuity of the activity in the oil refinery during our 2012 sampling campaign disrupted the microbial diversity of the EDB mat, leading to a divergent microbial community profile (Fig. 2). Excluding these samples, the microbial community composition was relatively similar in both sites (Bray–Curtis similarity index: 71.44%) and stable over the three sampling campaigns. Bacteria strongly predominated the prokaryotic community with on average 98.67 ± 0.48% of the 16S rRNA reads whereas a maximum of 2.02% of the 16S rRNA reads was affiliated to *Archaea*. *Bacteroidetes*, representing on average 18.64% of the 16S rRNA reads, *Cyanobacteria* (8.32%), *Alphaproteobacteria* (12.53%), including *Rhodobacterales* order, *Desulfobacterota* (10.82%) including the *Desulfobacterales* order and, *Gammaproteobacteria* (18.10%) were detected as major linages of the microbial mats, representing together up to 68% of the 16S rRNA genes identified in the metagenomes. In addition, members of *Chloroflexi* (4.26%), *Campylobacterales* (1.54%), *Patescibacteria* (3.40%), and *Verrucomicrobia* phyla (2.52%) were also detected in all samples but in minor proportions (Fig. 2).

### Metagenome assembled genomes of the microbial mat and mercury cycling populations

**Fig. 3:**
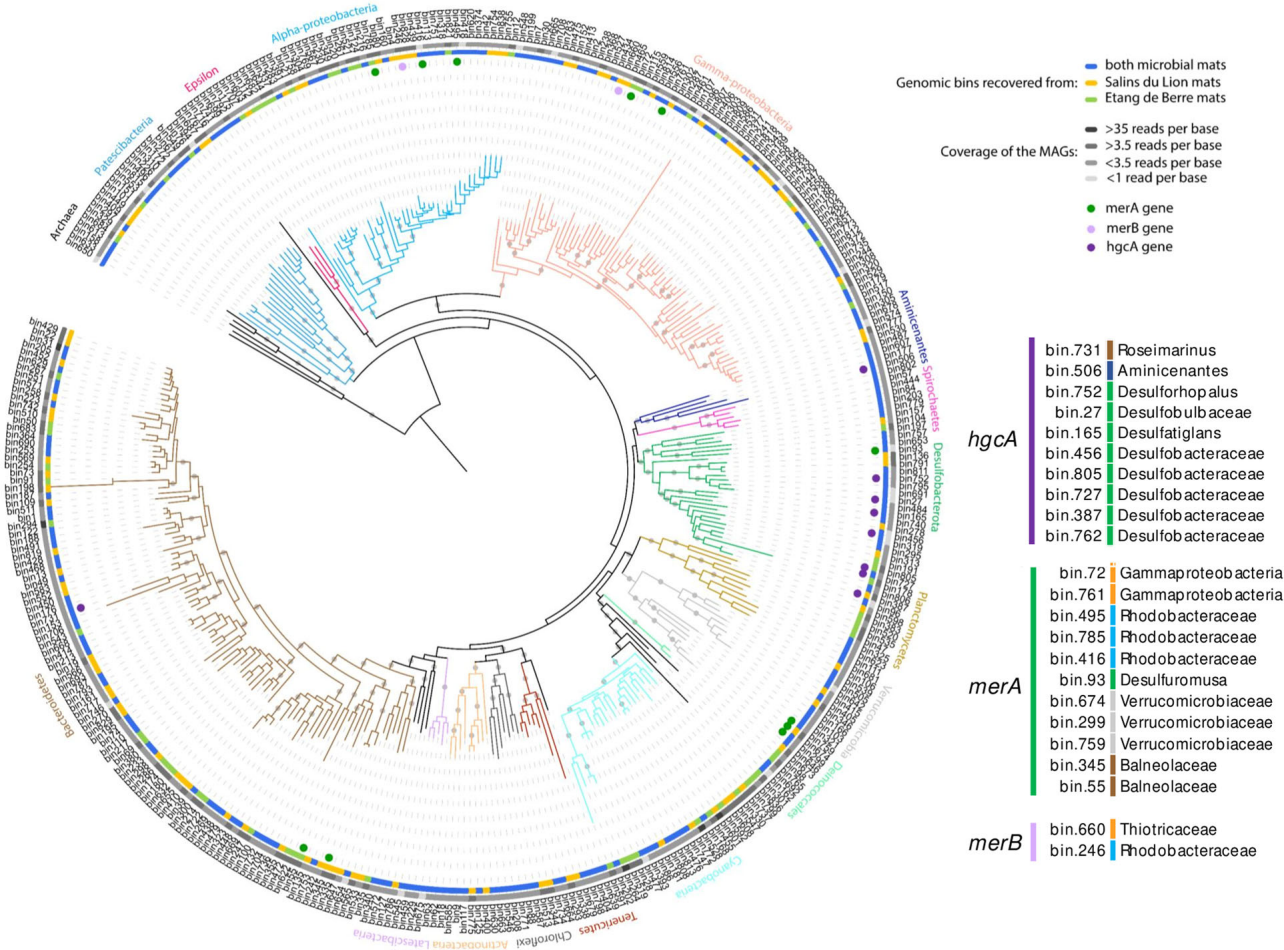
Phylogenomic tree of the 407 high- to medium-quality genomic bins recovered from the microbial mat metagenomes. The tree was constructed on the concatenated alignment of ribosomal protein genes. Only genomic bins with more than 50% of the ribosomal protein genes were included in the analysis. The tree was rooted with the archaeal bins. Branches are color coded as in Fig. 1. Purple, green, and pink dots indicate the detection of *hgcA*, *merA*, and *merB* genes in the bins, respectively. Colored circle at the edge of the branches indicates the microbial mat of origin of the bins with, in yellow and green, bins recovered only from SL and EDB samples, respectively, and in blue, bins recovered from both microbial mat samples. The second circle in shades of gray indicates the average coverage of the MAGs. Only bootstraps >0.8 were represented by gray points.

Binning of the contigs obtained after coassembly of all metagenomic reads resulted in the recovery of 407 high- to medium-quality MAGs (completeness > 50%, contamination < 5%). Taxonomic affiliation of these MAGs, inferred from 16S rRNA genes and ribosomal genes phylogenetic analysis (Fig. 3) indicated that MAGs from both dominant and rare lineages of the microbial mats were reconstructed. *Bacteroidetes* (72 MAGs), *Cyanobacteria* (25 MAGs), *Alphaproteobacteria* (42 MAGs), *Gammaproteobacteria* (68 MAGs), *Desulfobacterota* (26 MAGs), *Patescibacteria* (19 MAGs), and *Verrucomicrobia* (14 MAGs) were the most represented phyla (Fig. 2).

**Fig. 4:**
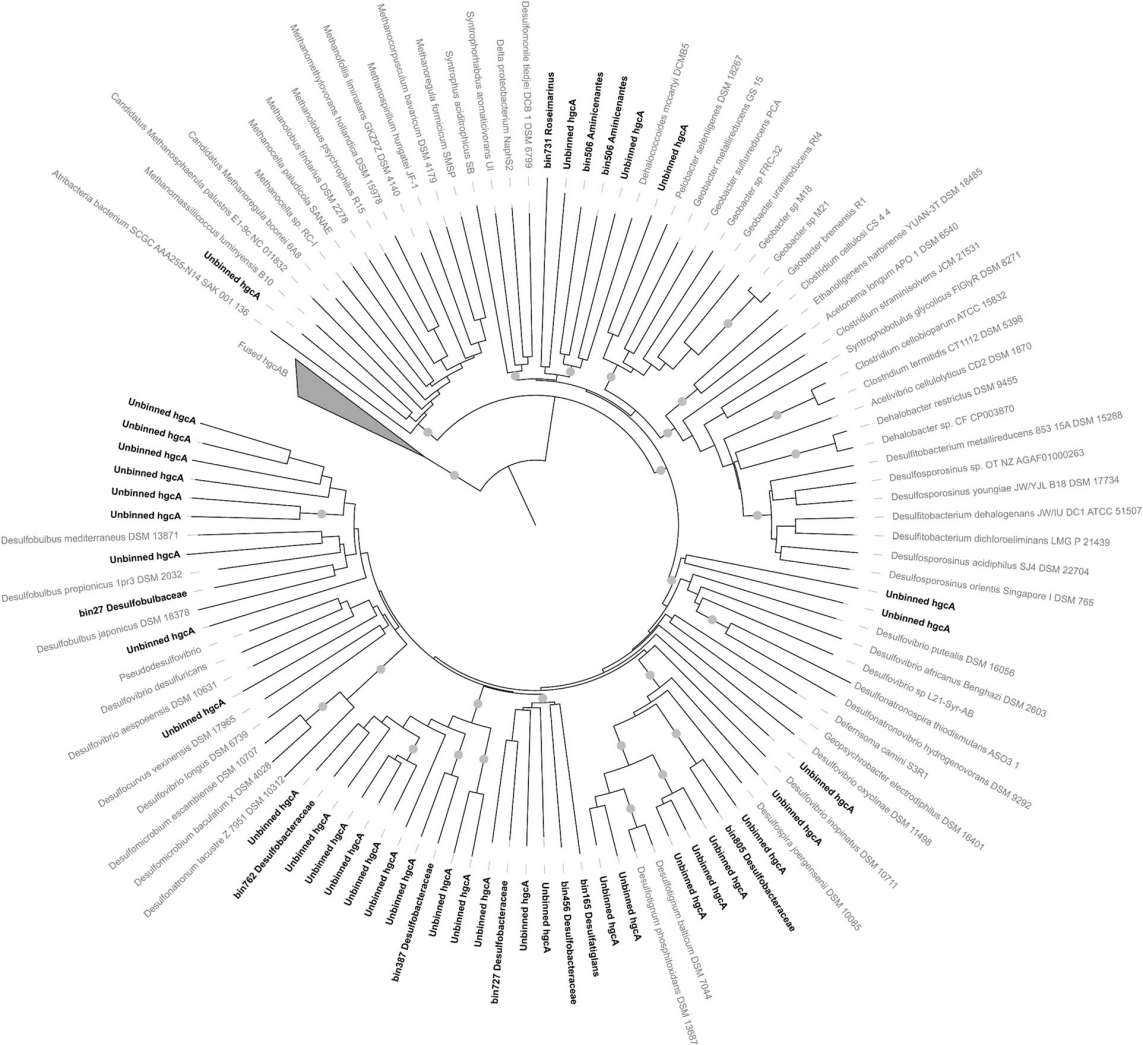
Phylogenetic tree of the *hgcA* genes. Binned and unbinned sequences identified in the metagenomic dataset are in bold. The tree was rooted with the fused *hgcAB* gene sequences. Bootstraps >0.8 are represented by gray points.

Mercury methyltransferase gene *hgcA*, mercury reductase gene *merA*, and alkylmercury lyase gene *merB* were screened in the metagenomic datasets. Up to 45 different *hgcA* genes (binned and unbinned) were identified in the metagenomic dataset (Fig. 4). Based on taxonomic analysis of *hgcA* sequences, 82% of these genes were affiliated to the *Desulfobacterota* phylum. Binning of the contigs recovered 10 MAGs with *hgcA* gene, representing most of the *hgcA* gene diversity identified in the samples, with 8 MAGS affiliated to *Desulfobacterota* (*Desulforhopalus*, *Desulfobulbaceae*, *Desulfatiglans,* and *Desulfobacteraceae* (Fig. 4). In addition, one MAG was affiliated to *Roseimarinus* (*Bacteroidetes*) and another one to the *Aminicenantes* phyla (Table 1 and Figs. 3 and 4). All of the binned *hgcA* genes were followed on their contigs by the *hgcB* gene. Unbinned *hgcA* sequences without binned references were related to archaeal methanogen (1 sequence), *Geobacter* (1 sequence), and *Desulfovibrio* (3 sequences) groups (Fig. 4).

**Table 1:** Detailed description of the MAGs with mercury transformation genes recovered in the microbial mats.

Bin ID	Taxonomy	Complet. (%)	Conta. (%)	16S rRNA gene	Relative abundance (av. coverage)	Mercury cycling gene	Number of ORF	Number of gene with predicted protein	Number of gene expressed
Bin731	*Roseimarinus*	87.28	5	Yes	1.56	*hgcAB*	3827	484	2830
Bin506	*Aminicenantes*	57.45	2.65		1.03	*hgcAB*	4462	447	1755
Bin752	*Desuforhopalus*	86.41	1.52	Yes	1.16	*hgcAB*	3543	526	2810
Bin27	*Desulfobulbaceae*	65.67	2.11		0.98	*hgcAB*	2613	350	1975
Bin165	*Desulfatiglans*	98.06	2.6	Yes	1.12	*hgcAB*	3733	385	3428
Bin456	*Desulfobacteraceae*	66.32	2.82		0.99	*hgcAB*	2566	323	1935
Bin805	*Desulfobacteraceae*	91.61	4.27	Yes	4.20	*hgcAB*	8173	1058	7361
Bin727	*Desulfobacteraceae*	88.42	4.2		1.10	*hgcAB*	5617	780	4529
Bin387	*Desulfobacteraceae*	54.08	1.61		9.42	*hgcAB*	3064	424	2886
Bin762	*Desulfobacteraceae*	53.75	1.79		1.13	*hgcAB*	3546	502	2713
Bin761	*Gammaproteobacteria*	89.00	3.05		1.34	*merA*	3563	424	2986
Bin495	*Rhodobacteraceae*	50.23	4.85		1.29	*merA*	1966	273	1149
Bin785	*Rhodobacteraceae*	93.64	2.7	Yes	17.82	*merA*	3600	487	1072
Bin416	*Rhodobacteraceae*	50.01	1.08		0.80	*merA*	1791	215	855
Bin93	*Desulfuromusa*	83.87	2.1	Yes	0.79	*merA*	2580	337	2356
Bin674	*Verrucomicrobiaceae*	94.76	1.16		5.25	*merA*	3036	277	2223
Bin299	*Verrucomicrobiaceae*	52.00	0		1.98	*merA*	2124	178	111
Bin759	*Verrucomicrobiaceae*	97.47	4.76	Yes	2.48	*merA*	4343	438	2489
Bin345	*Balneolaceae*	90.16	2.55		19.83	*merA*	2935	327	2843
Bin55	*Rubrivirga*	97.27	1.37	Yes	33.86	*merA*	3708	348	399
Bin72	*Gammaproteobacteria*	96.88	1.16	Yes	3.76	*merA*	3027	302	2509
Bin660	*Thiotricaceae*	96.89	2.93	Yes	1.71	*merB*	3124	340	2371
Bin246	*Rhodobacteraceae*	52.53	4.55		5.63	*merB*	2507	399	1824

**Fig. 5:**
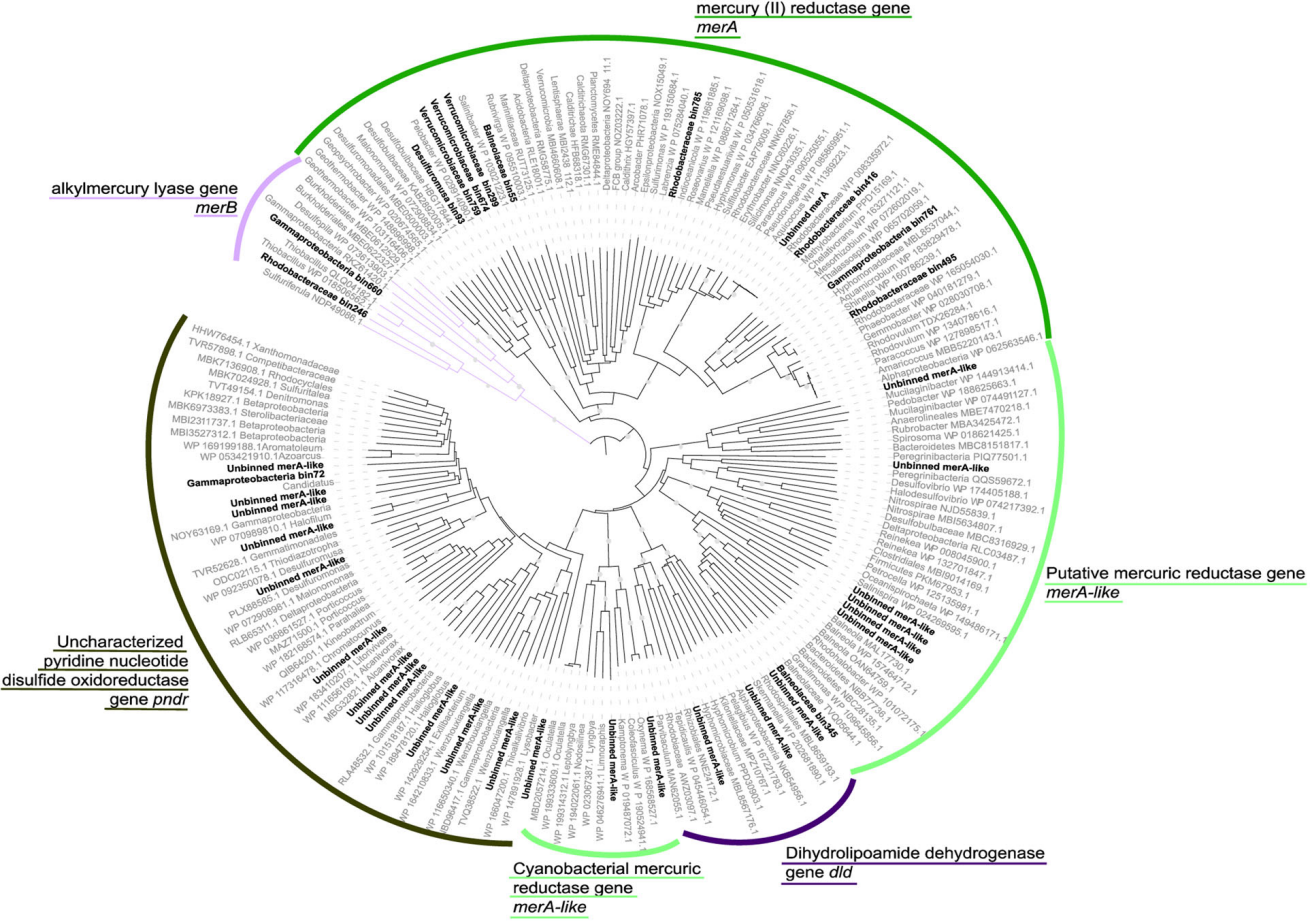
Phylogenetic tree of the putative mercury reductase gene (*merA*). Binned and unbinned sequences identified in the metagenomic dataset by the bioinformatic pipeline are in bold. The tree was rooted with alkylmercury lyase gene (*merB*) sequences. Due to sequence proximity with other genes of the pyrimidine nucleotide disulfide oxidoreductase family (*pndr*, *dld*), multiple incorrect assignments to putative *merA* genes were identified. Bootstraps >0.8 were represented by gray points.

After phylogenetic analysis of the *merA* genes recovered from the metagenomes, a total of 20 putative *merA* genes (10 *merA* and 10 *merA*-like) were identified, whereas 16 sequences were found to be misannotated by the bioinformatic pipelines (uncharacterized *pndr* and *dld* genes in Fig. 5). Furthermore, analysis of the amino acid sequences confirmed the presence of essential cysteines in MerA protein sequences^20^ whereas these amino acid signatures were not detected in MerA-like protein sequences. Nine of the 10 *merA* genes and 1 of 10 *merA*-like genes were found in the recovered MAGs. These MAGs were affiliated to *Gammaproteobacteria*, *Rhodobacteraceae* (*Alphaproteobacteria*), *Desulfuromusa* (*Desulfobacterota*), *Verrucomicrobiaceae*, and *Bacteroidetes* (*Balneolaceae* and *Rubrivirga*) (Table 1). In addition, unbinned *merA-like* genes without binned close representative were affiliated to *Cyanobacteria* and *Peregrinibacteria* (Fig. 5).

Only two *merB* genes were identified in the metagenomic dataset. These genes were identified in one *Thiotrichaceae* (*Gammaproteobacteria)* MAG (bin660) and one *Rhodobacteraceae* MAG (bin246), but none of these MAGs also included the *merA* gene (Fig. 5).

Coverage of the MAGs was used as a proxy for the relative abundances of the corresponding populations (Table 1). Overall, the average coverage of the 407 recovered MAGs was 3.5 reads per base in the contig, with a median value of 1.62 and a maximum of 203.12 for a cyanobacterial MAG. Most of the MAGs with *hgcA*, *merA,* or *merB* genes (62.5% of the MAGs) were recovered with a coverage lower than the average coverage (Table 1). Nonetheless, bin805 and bin387 with *hgcAB* genes, bin785, bin674, bin345, bin55, and bin72 with *merA* gene, and bin246 with *merB* gene were recovered with a coverage above the average coverages of all recovered MAGs (Table 1). In addition, coverage of unbinned contigs with *hgcAB* or *merA* genes was lower or similar to the binned contigs with coverage <1.5 and 4.5 reads per base for unbinned contigs with *hgcAB* and *merA* respectively.

### Expression profile of mercury transformation genes

**Fig. 6:**
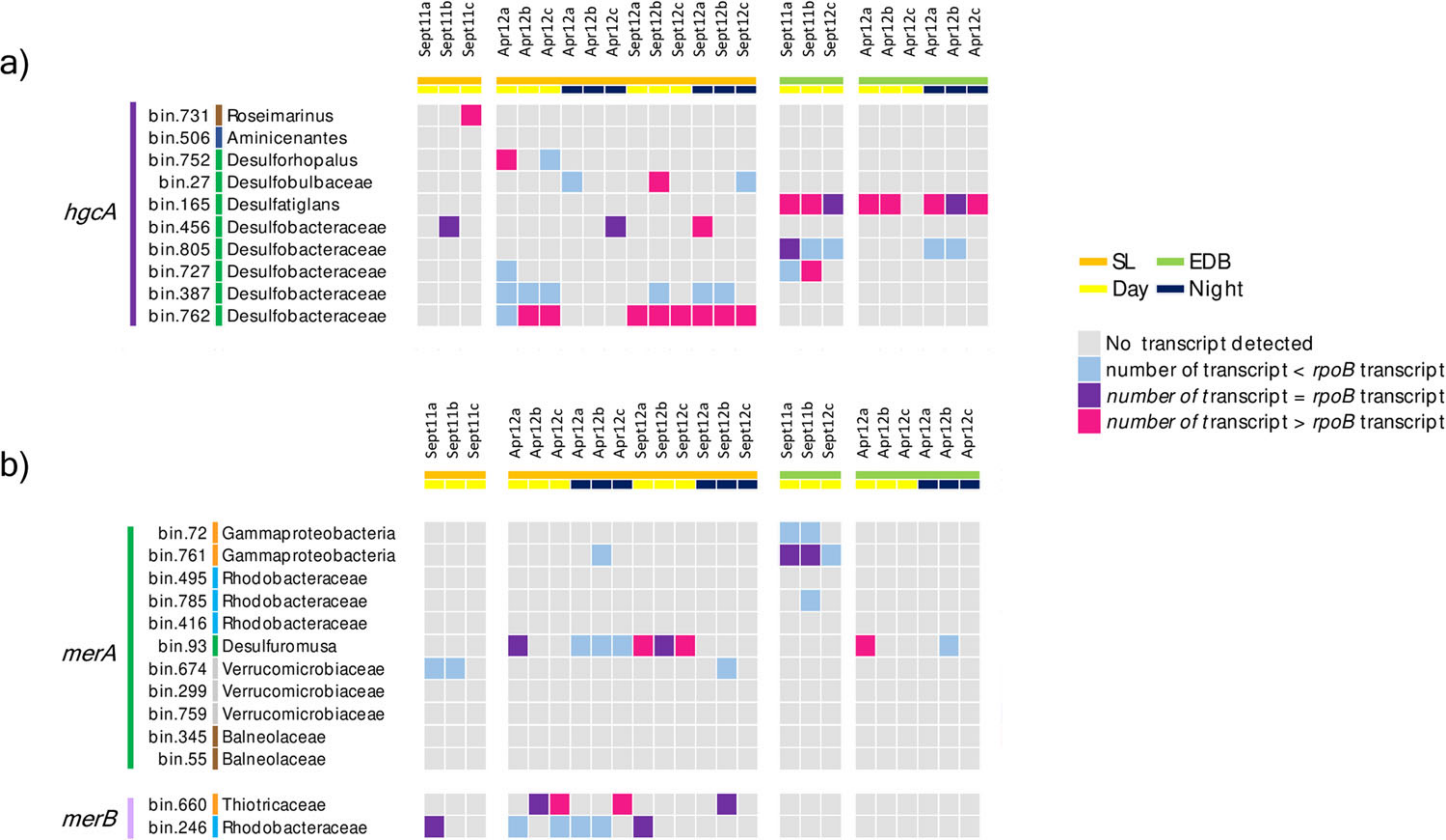
Expression profiles of a *hgcA*, b *merA* and *merB* genes over time in the genomic bins. Expression level was compared to the expression level of *rpoB* identified in each bin. Genomic bins are color coded as in other figures.

A MAG-centric metatranscriptomic approach was carried out to evaluate expression of mercury transformation genes (*hgcA, merA* and *merB*) in the microbial mats (Fig. 6). No mercury cycling gene was found to be expressed in all sampling dates and no clear pattern of expression could be identified between fall and spring periods or day and night, despite the decrease of oxygen concentration and redox potential during the night due to oxygen consumption and reduced photosynthetic activity (Fig. 1).

Transcripts of *hgcA* genes were detected in both microbial mats (Fig. 6a). Except for the *Aminicenantes*, expression of *hgcA* was detected for all populations in at least one sample. *hgcA* was strongly expressed (number of *hgcA* transcripts > number of *rpob* transcripts) by the *Desulfatiglans* (bin165) population in the EDB samples whereas a strong expression of *hgcA* by a *Desulfobacteraceae* (bin762) population was identified in most of the SL samples (Fig. 6a).

Transcripts of *merA* gene were identified in both EDB and SL microbial mats (Fig. 6b). Transcription of *merA* was identified for four populations: *Gammaproteobacteria* bin761, *Rhodobacteraceae* bin785, *Verrucomicrobia* bin674, and *Desulfuromusa* bin93. Only the *Desulfuromusa* bin93 population expressed *merA* at higher level than the housekeeping gene *rpoB*. Finally, although *merA* gene was detected in *Balneolaceae*, no *merA* transcript of *Balneolaceae* was identified in the metatranscriptomic dataset (Fig. 6b).

Transcription of *merB* genes was only detected in SL samples. All *merB* transcripts were assigned to the *Thiotrichaeae* bin660 and the *Rhodobacteraceae* bin246 populations (Fig. 6b). When detected, the transcription level of *merB* gene in bin660 was higher or similar to the expression level of *rpoB* gene while expression of *merB* in bin246 was lower or similar to *rpoB* expression.

### Metabolic pathways expressed by mercury cycling populations

**Fig. 7:**
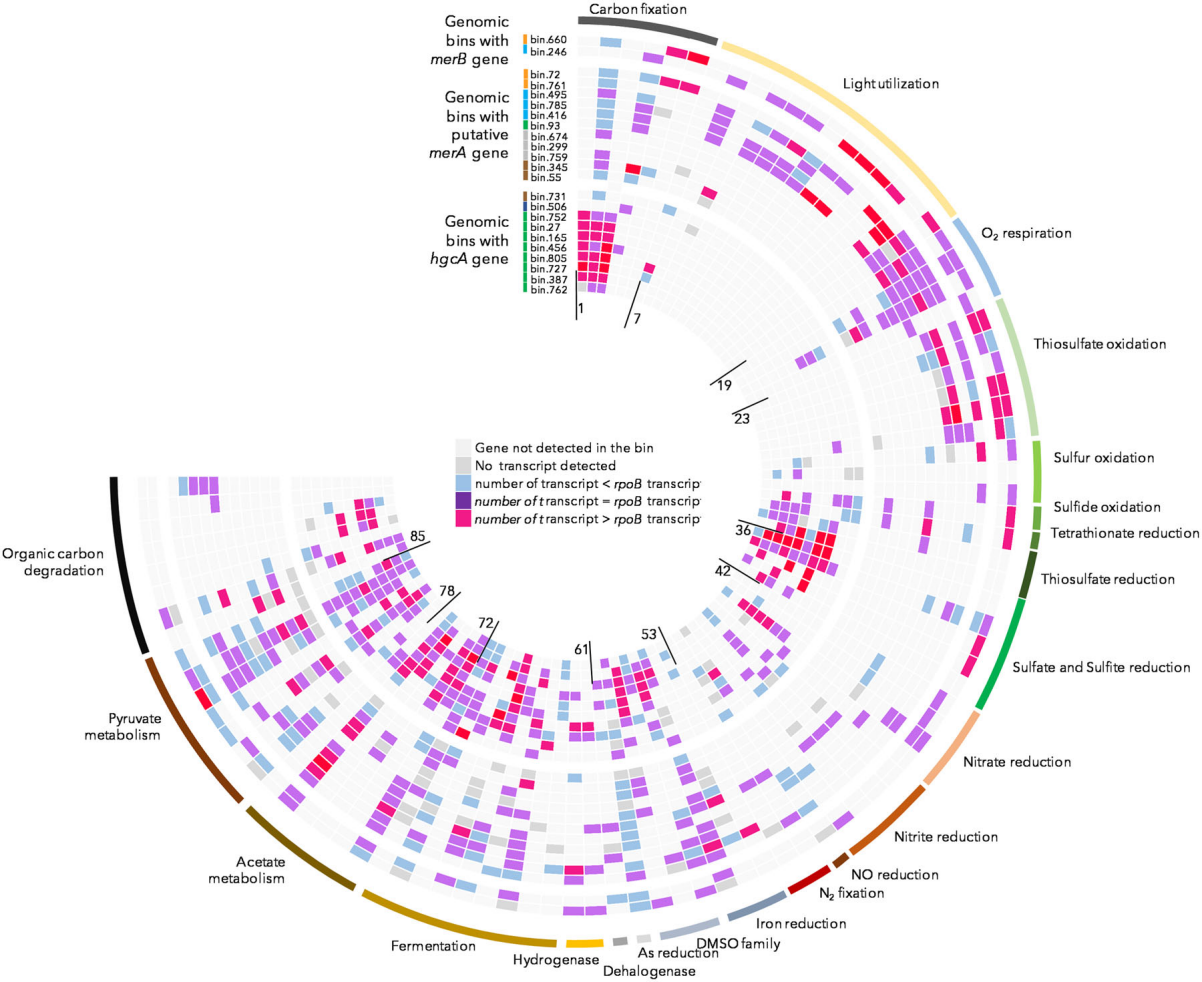
Gene expression profile of genomic bins potentially involved in mercury transformations. For each bin, all transcripts identified per gene over the different sampling periods were summed, then the summed number of transcripts per gene was compared to the number of *rpoB* gene transcripts to determine the relative expression level. List of the genes and associated dataset is provided in Supplementary Data 1.

Genes expression profile of mercury cycling populations was investigated to determine their putative metabolic activities within the microbial mats (Fig. 7 and Supplementary Data 1). Although the expression profile of each bin depended on its completeness, an average of 2409 ± 1470 genes per bin was found to be expressed over the different seasons and daytime of the study (Table 1).

The *Desulfobacterota* populations with *hgcAB* genes expressed numerous metabolic pathways including sulfate (*sat*, *aprAB*, *dsrABC*), tetrathionate (*otr*), thiosulfate/polysulfide (*phs*-*psrABC*), and sulfur reduction (*dsrE*) genes. Transcription of nitrate, nitrite, and nitric oxide reduction (*narG*, *nrfA,* and *norQ*) and nitrogen fixation genes was also largely detected in *Desulfobacterota* populations (Fig. 7). Transcripts of iron reduction genes *omcA*/*mtrC* were detected in two populations (bin805 and bin387) whereas sulfur oxidation (SQR) and cytochrome *C* oxidase genes were expressed by the *Desulforhopalus* (*Desulfobulbaceae)* bin752 population that also expressed nitrate and nitrite reduction genes. Expression of key enzymes of the Wood–Ljungdahl pathway and subsequent acetate and pyruvate metabolism was also detected for all *Desulfobacterota* populations. Genes coding hydrogenases and malate, lactate, alcohols, and formate dehydrogenases were also strongly expressed among *Desulfobacterota* populations. In addition, transcripts of 2-haloacid dehalogenase and trimethylamine-*N*-oxide reductases (*yedYZ* and *torC*) were also detected for half of the *Desulfobacterota* populations. Hydrocarbon degradation genes (*assA*, *bssA*, *badA*) were expressed in all *Desulfobacterota* populations recovered from EDB. The *Roseimarinus* (bin731) population with *hgcAB* genes expressed genes of tetrathionate/nitrate and nitrite reducing cytochromes (*otr*, cytochrome *c*-552, and *nrfAH*), bacteriochlorophyll synthesis (*bhsC*), and pyruvate metabolism (Fig. 7). The *Aminicenantes* (bin506) population with *hgcAB* genes also expressed nitrate and nitrite reduction genes (*napA*, *otr*, and *nrfAH*) as well as alcohol dehydrogenase and pyruvate metabolism genes. Alkyl succinate synthase gene (*assA*) involved in hydrocarbon degradation was identified in bin506 but not expressed (Fig. 7).

The microbial populations with *merA* gene expressed a large variety of metabolic pathways including light utilization for members of the *Bacteroidetes*, *Alpha*- and *Gammaproteobacteria*, sulfur (DsrE), sulfide (SQR), and thiosulfate oxidation (Sox operon) for the *Alpha*- and *Gammaproteobacteria*, thiosulfate, nitrate (*narG*), and nitrite (*nrfA*) reduction for *Desulfobacterota*. Iron reduction encoding genes (*omcA*/*mtrC*, and predicted CXXCH cytochrome) were also expressed by the *Desulfobacterota* and the *Gammaproteobacteria* bin761 populations. *Gammaproteobacteria* bin72, which lacked light utilization genes, expressed the Calvin–Benson–Bassham cycle, a complex sulfur oxidation pathway (*sox*, *SQR*, *dsrE*, *fccA*), as well as nitrite reductase and nitrogen fixation genes (*nif*). Genes coding for nitrate (*narG*) and arsenate reductases (*asrA*), hydrogenase, halogen, urea, and ethanolamine utilization were also expressed by a large fraction of the *merA-*bearing populations (Fig. 7).

For the *merB*-bearing *Thiotrichaeae* (*Gammaproteobacteria*) bin660 population, a strong expression of the Rubisco, cbb-3 type cytochromes, *sox* operon, and the oxidative-type *dsrAB* genes was detected. Expression of nitrate reducing genes (*narG* and *otr*) and hydrogenases was also observed. The *Rhodobacteraceae* (*Alphaproteobacteria*) bin246 population with *merB* gene strongly expressed light-harvesting complex and photosynthetic reaction center genes (*pufLM*), nitrate reductase genes (*narABG*), and the Sox operon. The lower level of transcription was also detected for dehalogenase and arsenate reductase genes (Fig. 7).

## Discussion

To better understand the fate and transformations of mercury compounds in coastal environments, the microbial community composition, function, and potential activity of two shallow microbial mats exposed to strong mercury contamination were investigated in detail. The full thickness of the mats was collected, including the oxic and anoxic layers, allowing the simultaneous analysis of both aerobic and anaerobic lineages and metabolisms. Additionally, sampling was carried out at different seasons to account for temporal variability. At the genus level, the microbial community composition was similar in both EDB and SL microbial mats (Fig. 2, Bray–Curtis similarity index: 71.44%), and aligned with microbial mat communities identified in other marine and brackish ecosystems^16,21,22^, allowing our results to be reasonably extended to similar environmental contexts.

To evaluate mercury methylation in the microbial mats, *hgcA* genes and transcripts were investigated. The frequency of *hgcA* genes identified in the metagenomic dataset (Fig. 4) was similar to previous reports on marine polluted sediments^23^, indicating a strong potential for methylation of mercury in microbial mats. All *hgcA* genes identified in genomic bins were followed by *hgcB* gene in the contigs, supporting the metabolic potential for mercury methylation^24^. Mercury methylation genes were identified in genome of populations related to *Roseimarinus* (*Bacteroidetes*) and the uncultivated phyla of *Aminicenantes*, supporting previous metagenomic mining in marine environments^7^. However, phylogenetic analysis of *hgcA* gene and transcripts as well as taxonomy of the *hgcAB*-bearing MAGs indicated that members of the *Desulfobacterota* represented most of the mercury methylator diversity in these coastal microbial mats (Figs. 4 and 6a), which is consistent with the sulfate and sulfide rich conditions of the mats (Fig. 1). *Desulfobacteraceae* bin387 and bin805 populations were recovered with high coverages (>4 reads per base; Table 1), suggesting that these populations are quantitatively important members of the microbial mats. However, the large majority of the *hgcA* transcripts were related to less abundant hydrocarbon degrading *Desulfatiglans* bin165 population in the Etang de Berre mat and a different *Desulfobacteraceae* population (bin762) in Salins du Lion. These results suggest that although various populations have the potential to methylate mercury, only few populations expressed the genes, supporting similar observation in thawed permafrost ecosystem^6^ and that most of the mercury methylation activity might be carried out by minority to rare members of the microbial community, challenging correlation analyses between methylmercury concentration and relative abundance of potential mercury methylators. This finding also supports previous examples for microorganisms, and more particularly *Desulfobacterota* members, of numerical low abundance that can have an impact on ecosystem functioning^25^.

A large variety of metabolic pathways were expressed by mercury methylators, spanning several biogeochemical cycles and extending known ecological functions of these microorganisms (Fig. 7). These metabolic activities are probably not attributable to a single bacterium but distributed between the different members of each population, with each member exploiting different environmental microniches. As expected from previous results based on samples of similar environmental conditions, the dissimilatory sulfate reduction pathway was one of the most expressed energetical pathway in mercury methylator populations^2,26–28^. However, we also detected transcripts of tetrathionate and thiosulfate/polysulfide reductases in 60% of *Desulfobacteraceae* populations, suggesting that part of the mercury methylators might gain energy from additional sulfur sources. Interestingly, transcripts of cytochrome *c* oxidase and sulfide quinone oxidoreductase (SQR) enzymes were identified in the *Desulfobulbaceae* population, supporting a sulfide/sulfur oxidation metabolism, potentially coupled to nitrate reduction through the expression of *narG* gene, as previously proposed^29^. This result suggests that mercury methylation might not be limited to low redox and anaerobic conditions, but could also be carried out in oxic to suboxic conditions by some *Desulfobulbaceae* populations, potentially extending the distribution of environmental niches prone to methylmercury production.

Transcripts analysis revealed a major involvement of mercury methylators in nitrogen cycling with expression of nitrate reduction and/or denitrification genes for all recovered populations (Fig. 7), as previously proposed^30,31^. Expression of genes involved in iron, halogenated and methylated compounds utilization and various fermentation was also detected, suggesting that some methylmercury producers might thrive on numerous additional metabolism than previously suspected. In addition, genes associated with syntrophic metabolism (hydrogenases, formate dehydrogenases)^32^ were also expressed by *Desulfobacterota* populations, supporting a potential role of syntrophic interactions in mercury methylation^33^. Together these results expanded the environmental niches and the known metabolic flexibility of mercury methylators.

As toxic methylmercury and mercury accumulation might be mitigated by demethylation and reduction, through the activity of organomercury lyase (MerB) and mercury reductase (MerA) enzymes respectively^11^, diversity of *merB* and *merA* genes were also assessed. Based on *merA* genes phylogeny and taxonomy and coverage of the MAGs, the diversity (number of different lineages) and relative abundance (average coverage) of mercury reducers were larger than those of mercury methylators (Fig. 3 and Table 1). Mercury reducers detected in the microbial mats included known lineages of mercury reducers such as members of the *Gammaproteobacteria*, *Rhodobacteraceae*, and *Bacteroidetes*^11^. *Rhodobacteraceae* and *Bacteroidetes* populations were identified as dominant members of the microbial mats (Fig. 2) but no or very little expression of *merA* was detected for these lineages (Fig. 6). Most of the *merA* transcripts were assigned to *Gammaproteobacteria* (bin761) and *Desulfuromusa* (bin93) populations with low coverage (Table 1), suggesting that mercury detoxification activity of abundant microorganisms might be lower than those of rare lineages.

Genetic composition and expression profile of the *Desulfuromusa* bin93 population (Fig. 7) was consistent with the strict anaerobic metabolism of the *Desulfuromonadales* members^34^, suggesting that although *merA* is generally identified in aerobic lineages^35,36^, most of the mercury reduction might be carried out by anaerobic lineages in the anoxic layers of microbial mats and sediments. This results suggest a potential cycle of mercury methylation, demethylation, and reduction in anoxic part of the microbial mat, as previously suspected in other anoxic environments^36^. Interestingly, *merA* gene was also identified in three *Verrucomicrobiaceae* populations with the genetic composition and the expression profile consistent with an anaerobic metabolism based on fermentation (Fig. 7). The *merA* gene of these populations branched at the base of the phylogenetic tree without known references but in proximity with *Desulfuromonadales* sequences (Fig. 5), indicating that these *Verrucomicrobiaceae* populations may represent a new group of mercury reducers under anoxic conditions. Expression of *merA* gene was detected for one of the *Verrucomicrobiaceae* population (Fig. 6), supporting their role in mercury detoxification. Together, detected activities of mercury reducer populations were diverse, spanning sulfur, nitrogen and iron cycles and phototrophic, autotrophic and heterotrophic lifestyles, indicating a large metabolic flexibility in aerobic and anaerobic mercury detoxifying populations.

None of the recovered MAG with *merA* gene also included the organomercury lyase gene (*merB*). However, *merB* gene was identified in two MAGs with high completeness (96.89%), detected exclusively in the Salins du Lion mats, and affiliated to members of the *Rhodobacteraceae* and *Thiotrichaceae* with sulfur-oxidizing activity. Although this might be due to the incompleteness of our MAGs, this result is consistent with the low frequency of *merA* and *merB* gene cooccurrence in public microbial genomes^11^. Furthermore, mining of the available genomes in IMG database for *merA* and *merB* genes highlighted that over the 4530 genomes with *merB* gene, only 34% (1545) also include *merA*, supporting the occurrence of *merB* alone in our MAGs and recent observations^20^. Both *Rhodobacteraceae* and *Thiotrichaceae* populations were found to express *merB* (Fig. 6), indicating an active methylmercury detoxification process in SL microbial mat. Together our results suggest that methylmercury cleavage and mercury reduction were dissociated in the microbial mats, with each reaction being carried out by taxonomically different lineages. The accumulation of mercury is detrimental for the cells, therefore additional *merA*-independent mechanism for mercury export or detoxification should occur in these populations. Various biotic and abiotic *merA*-independent detoxification process have been proposed^20,37^, with notably the reduction of HgII to Hg0 in photomixotrophic microorganisms, providing a plausible mechanism of mercury detoxification for the *Rhodobacteraceae* bin246 population that strongly expressed phototrophic and mixotrophic genes.

The presence of a long-term hydrocarbon contamination in Etang de Berre is the main environmental feature that distinguishes the two habitats sampled in this study^15^ (Fig. 1). Our 16S rRNA gene analysis indicated that the microbial community composition at the genus level was similar in the two habitats. However, the genome centric approach indicated that up to 162 different populations (40% of the MAGs, Fig. 3) were recovered from only one habitat, suggesting a modification of the microbial community composition at the population level rather than at the genus level. Consistent with the alkane and PAH contamination in Etang de Berre, all MAGs with the potential for mercury methylation identified in this mat (bin165, bin506, bin727, bin805) were found to encode and express anaerobic hydrocarbon degradation genes (Benzoyl-CoA reductase or Aryl/benzylsuccinate synthase, annotated 88 and 89 in Fig. 7) except for the *Aminicentantes* bin506 for which no transcripts were identified (Fig. 7). By contrast, none of the MAGs from Salins du Lion encoded such genes. This result might indicate that hydrocarbon could structure the mercury methylator community by selecting lineages with hydrocarbon degrading metabolism. Although additional experiments are required, the absence of methylmercury degraders in EDB could also be a consequence of the hydrocarbon pollution in this habitat. By affecting both methylmercury producer and degrader diversity, the presence of hydrocarbons might have critical consequences for the methylmercury fate in hydrocarbons polluted environments.

The microbial mats analyzed in detail in this study provided an outstanding opportunity to investigate microbial populations controlling mercury toxicity at the foundation of the food chain. Our genetic and transcriptomic characterization of involved microbial populations extended the taxonomic and functional diversity of mercury cycle lineages, and emphasize the major roles of minority and rare mercury transformers in sulfur but also carbon, nitrogen, and iron biogeochemical cycles. The simultaneous detection of genes and transcripts of both mercury methylation and detoxification pathways indicated that mercury transformations are entangled in brackish biofilms, with potential cycles of methylation and demethylation under both anoxic and suboxic conditions.

## Methods

### Sample collection and nucleic acid extraction

Two coastal microbial mats developing in neighboring brackish lagoons, located at Berre l’Etang (EDB) and Salins du Lion (SL), a bird reserve located 5 km away from EDB were investigated (Fig. 1). Physical (temperature, pH, salinity, irradiance) and chemical (hydrocarbon content, metal, sulfate, H_2_S, dissolved oxygen, total and organic carbon and total nitrogen concentrations, redox potential) parameters have been previously published for both microbial mats and underlying sediments^19^. Sediments under both microbial mats are strongly contaminated by heavy metals, with mercury concentration averaging to 17.8 ± 4.1 μg of Hg per gram^19^. The major environmental parameter that distinguishes the two microbial mats is the presence of hydrocarbons, including alkanes and polycyclic aromatic hydrocarbons, caused by oil refinery contaminated rainwaters discharges in EDB^15^. Microbial mat samples were collected in triplicate in September 2011, April 2012, and September 2012. In September and April 2012 microbial mats were sampled during both daytime (4 PM) and night-time (4 AM), leading to a total of 30 microbial mat samples. Samples were stored in cryotubes and immediately submerged in liquid nitrogen for fast-freezing. Back in laboratory, samples were stored at −80 °C until nucleic acids extraction. Nucleic acids (DNA and RNA) were extracted from all samples using the RNA PowerSoil^TM^ Total RNA Isolation kit (Qiagen, Hilden, Germany) coupled with the Allprep DNA/RNA mini kit and the RNaseFree DNase Set (Qiagen). RNA quality was checked using RNA nanochip on a Bioanalyzer 2100 (Agilent, Santa Clara, CA, USA) and absence of DNA contamination within RNA samples was confirmed by PCR amplification using RNA as a template. mRNA were enriched using Ribo-Zero™ Magnetic Kit (Bacteria), following the manufacturer’s instructions. The RNA was then immediately converted to cDNA using M-MLV Reverse Transcriptase and RNase OUT^TM^ (Invitrogen, Carlsbad, CA, USA) with 1 µl of purified mRNA according to the manufacturer’s instructions.

### Metagenomic library preparation, sequencing, and analysis

Metagenomes were constructed for all 30 samples using the BioScientific PCR-free kit following the manufacturer’s protocol while metatranscriptomes of each sample were prepared using the TruSeq Stranded mRNA sample prep kit (Illumina, San Diego, CA, USA) following the manufacturer’s instructions. Metagenomes and metatranscriptomes were sequenced using Illumina HiSeq (2 × 100 bp) platform at the Genotoul sequencing facility leading to a total 670 Gb of sequences (370 GB of metagenomes and 300 GB of rRNA-depleted metatranscriptomes). Datasets were quality filtered using the Trimmomatic v.0.39 tool^38^, keeping both R1 and R2 reads when reads overlapped. The 16S rRNA reads were isolated from the metagenomic reads using REAGO 1.1 (ref. ^39^), and taxonomic assignments were performed with Mothur^40^ using RDP classifier (cut-off: 80) against Silva database release 138 as reference^41^. Since metagenomic 16S rRNA reads were only 100 bp long and spanned various regions of the 16S rRNA gene, taxonomic assignments were limited to the genus level and above.

### Binning and functional characterization

For MAGs reconstruction, all quality filtered sequences were pooled and co-assembled using MEGAHIT^42^, then coassembly was uploaded to IMG/MER platform for gene annotation^43^. Read coverage of the contigs was carried out using bwa-mem (http://bio-bwa.sourceforge.net), followed by the binning of the contigs longer than 2000 bp by MetaBAT-2 (ref. ^44^). The completeness and contamination level of the MAGs were then evaluated using CheckM^45^. Only MAGs with a contamination level under 5% and completeness above 50% were analyzed. Open reading frames (ORFs) were identified using Prodigal^46^ and compared against COG, Pfam, TIGRfam, and KEGG databases on IMG/MER platform, leading to 5.7 × 10^6^ genes with product name (58.96% of the genes). Since *hgcA* gene is poorly characterized on large public databases, we identified *hgcA* genes in the dataset using the publicly available *hgcA* hidden markov model profile and metabolisHMM^47^. In addition, all contigs with *hgcA* were manually inspected to detect the presence of the *hgcB* gene downstream of *hgcA*. For the *merA* and *merB* genes identification, erroneous annotations were identified in the KEGG database. Therefore, *merA* and *merB* were also identified separately with metabolisHMM using KOFAM K00520 for *merA* and a homemade hidden markov model of *merB* genes, filtered with an *e*-value threshold of *e*^−^^120^. To validate gene identifications, recovered amino acid sequences of *hgcA, merA,* and *merB* genes were aligned with reference sequences downloaded from NCBI using Clustal Omega^48^ and amino acid phylogenetic trees were generated using Fasttree 2 (ref. ^49^). Phylogenetic trees were visualized using iTol v.4 (ref. ^50^). When the gene sequence was complete, recovered MerA sequences were manually examined for the presence of cysteine pairs that are potentially essential for MerA activity^20^. In the absence of the amino acid signature the gene were defined as “merA-like”. Likewise, the presence of conserved amino acid signatures identified in functional MerB proteins^51^, including cysteines at position 96, 159, and 117 and aspartic acid at position 99, was also verified in recovered MerB sequences.

### Expression profile analysis

To determine gene expression levels of each gene identified in MAGs, all metatranscriptomic reads passing quality filtration were mapped against all open reading frames identified in MAGs using bwa-mem. Due to rRNA depletion step malfunction, the September 2012 metatranscriptomes of EDB microbial mats were removed from the analysis. For each MAGs, the coverage value of genes of interest was normalized to the coverage of the RNA polymerase subunit B (*rpoB*) gene to discriminate between low and high expression level compared to housekeeping genes. Results were represented in heatmaps using the software environment R (v.4.0.3) and R Studio (v.1.3.1093).

### Reporting summary

Further information on research design is available in the Nature Research Reporting Summary linked to this article.

## Supplementary information

Supplementary Data 1

Reporting Summary

## Data Availability

Assembled metagenome data are available in IMG/MR (https://img.jgi.doe.gov/mer/) under the following accession numbers: 3300040774–3300040799. Coassembly is also available on IMG/MR under accession number 3300040801. Bins with mercury cycling genes were deposited in Figshare (10.6084/m9.figshare.15015303v2). Environmental metadata were previously published^15^. Nucleotide sequences are available in the NCBI Genbank database under Bioproject accession number SRP063590.

## Code availability

The merB gene HMM profile is available in Figshare (10.6084/m9.figshare.15015303.v2). Software version: REAGO 1.1, Mothur 1.44.3, Trimmomatic v0.39, Megahit 1.2.9, bwa 0.7.17, CheckM v.1.1.2, R 4.0.3 and R Studio 1.3.1093.
