# Supplementary material for: Transcriptomic evidence for versatile metabolic activities of mercury cycling microorganisms in brackish microbial mats
Source: NPJ Biofilms Microbiomes. 2021 Nov 19;7:83. doi: 10.1038/s41522-021-00255-y (PMC8605020; doi:10.1038/s41522-021-00255-y)
Supplement: Supplementary file 2 — Reporting Summary [file 41522_2021_255_MOESM2_ESM.pdf]

nature portfolio

Corresponding author(s): Vigneron Adrien

Last updated by author(s): Sep 20, 2021

Reporting Summary

Nature Portfolio wishes to improve the reproducibility of the work that we publish. This form provides structure for consistency and transparency in reporting. For further information on Nature Portfolio policies, see our Editorial Policies and the Editorial Policy Checklist.

Statistics

For all statistical analyses, confirm that the following items are present in the figure legend, table legend, main text, or Methods section.

n/a	Confirmed	
☐	☒	The exact sample size (*n*) for each experimental group/condition, given as a discrete number and unit of measurement
☒	☐	A statement on whether measurements were taken from distinct samples or whether the same sample was measured repeatedly
☒	☐	The statistical test(s) used AND whether they are one- or two-sided<br>*Only common tests should be described solely by name; describe more complex techniques in the Methods section.*
☒	☐	A description of all covariates tested
☒	☐	A description of any assumptions or corrections, such as tests of normality and adjustment for multiple comparisons
☒	☐	A full description of the statistical parameters including central tendency (e.g. means) or other basic estimates (e.g. regression coefficient) AND variation (e.g. standard deviation) or associated estimates of uncertainty (e.g. confidence intervals)
☐	☒	For null hypothesis testing, the test statistic (e.g. *F*, *t*, *r*) with confidence intervals, effect sizes, degrees of freedom and *P* value noted<br>*Give P values as exact values whenever suitable.*
☒	☐	For Bayesian analysis, information on the choice of priors and Markov chain Monte Carlo settings
☒	☐	For hierarchical and complex designs, identification of the appropriate level for tests and full reporting of outcomes
☐	☒	Estimates of effect sizes (e.g. Cohen's *d*, Pearson's *r*), indicating how they were calculated

Our web collection on statistics for biologists contains articles on many of the points above.

Software and code

Policy information about availability of computer code

Data collection	no software was used
Data analysis	The 16S rRNA reads were isolated from the metagenomic reads using REAGO 1.1, and taxonomic assignments were performed with Mothur using RDP classifier (cut-off: 80) against Silva database release 138 as reference .<br>For metagenome assembled genomes (MAGs) reconstruction, all quality filtered sequences were pooled and co-assembled using MEGAHIT, then co-assembly was uploaded to IMG/MER platform for gene annotation. Read coverage of the contigs was carried out using bwa-mem (http://bio-bwa.sourceforge.net), followed by the binning of the contigs longer than 2000 bp by MetaBAT-2. The completeness and contamination level of the MAGs were then evaluated using CheckM. Open reading frames (ORFs) were identified using Prodigal and compared against COG, Pfam, TIGRfam, and KEGG databases on IMG/MER platform. Since hgcA gene is poorly characterized on large public databases, we identified hgcA genes in the dataset using the publicly available hgcA hidden markov model profile and metabolisHMM. In addition, all contigs with hgcA were manually inspected to detect the presence of the hgcB gene downstream of hgcA. For the merA and merB genes identification, erroneous annotations were identified in the KEGG database. Therefore, merA and merB were also identified separately with metabolisHMM using KOFAM K00520 for merA and a homemade hidden markov model of merB genes, filtered with an e-value threshold of e-120. To validate gene identifications, recovered amino acid sequences of hgcA, merA and merB genes were aligned with reference sequences downloaded from NCBI using Clustal Omega and amino acid phylogenetic trees were generated using Fasttree 2. Phylogenetic trees were visualized using iTol v.4

For manuscripts utilizing custom algorithms or software that are central to the research but not yet described in published literature, software must be made available to editors and reviewers. We strongly encourage code deposition in a community repository (e.g. GitHub). See the Nature Portfolio guidelines for submitting code & software for further information.

Data

Policy information about availability of data

All manuscripts must include a data availability statement. This statement should provide the following information, where applicable:

- Accession codes, unique identifiers, or web links for publicly available datasets
- A description of any restrictions on data availability
- For clinical datasets or third party data, please ensure that the statement adheres to our policy

Assembled metagenome data are available in IMG/MR (https://img.jgi.doe.gov/mer/) under the following accession numbers: 3300040774-3300040799. Co-assembly is also available on IMG/MR under accession number 3300040801. Bins with mercury cycling genes were deposited in Figshare (10.6084/m9.figshare.15015303). Environmental metadata were previously published

Field-specific reporting

Please select the one below that is the best fit for your research. If you are not sure, read the appropriate sections before making your selection.

☐ Life sciences ☐ Behavioural & social sciences ☒ Ecological, evolutionary & environmental sciences

For a reference copy of the document with all sections, see nature.com/documents/nr-reporting-summary-flat.pdf

Ecological, evolutionary & environmental sciences study design

All studies must disclose on these points even when the disclosure is negative.

Study description	This study analyse the mercury cycling microorganisms and metabolism from two microbial mats sampled from a brackish lagoon in France
Research sample	Environmental microbial mats collected at shallow water depth
Sampling strategy	Microbial mats were sampled in triplicates at 3 different times, during day and night
Data collection	*Describe the data collection procedure, including who recorded the data and how.*
Timing and spatial scale	. Microbial mat samples were collected in triplicate in September 2011, April 2012 and September 2012. In September and April 2012 microbial mats were sampled during both daytime (4PM) and night-time (4AM), leading to a total of 30 microbial mat samples
Data exclusions	No data were excluded
Reproducibility	Samples were taken in triplicate and all replicate were analyzed separately
Randomization	Not relevant with our experimental procedure
Blinding	Not relevant with our experimental procedure

Did the study involve field work? ☒ Yes ☐ No

Field work, collection and transport

Field conditions	n September and April 2012 microbial mats were sampled during both daytime (4PM) and night-time (4AM)
Location	Microbial mats are located in Etang de Berre and Salin du Lion lagoons (France)
Access & import/export	Not relevant
Disturbance	Not relevant

Reporting for specific materials, systems and methods

We require information from authors about some types of materials, experimental systems and methods used in many studies. Here, indicate whether each material, system or method listed is relevant to your study. If you are not sure if a list item applies to your research, read the appropriate section before selecting a response.

Materials & experimental systems

n/a	Involved in the study
☒	☐ Antibodies
☒	☐ Eukaryotic cell lines
☒	☐ Palaeontology and archaeology
☒	☐ Animals and other organisms
☒	☐ Human research participants
☒	☐ Clinical data
☒	☐ Dual use research of concern

Methods

n/a	Involved in the study
☒	☐ ChIP-seq
☒	☐ Flow cytometry
☒	☐ MRI-based neuroimaging
